# Unexpected novel clade III type nitrous oxide-reducing bacteria from incubated lake sediments

**DOI:** 10.1093/ismeco/ycag194

**Published:** 2026-07-05

**Authors:** Siyu Wang, Fei Shui, Yiwen Zhou, Xiao Wang, Yuli Zeng, Yinghao Shan, Jining Li, Liguo Zhang, Kang Song, Fengchang Wu

**Affiliations:** National–Regional Joint Engineering Research Center for Soil Pollution Control and Remediation in South China, Guangdong Key Laboratory of Integrated Agro–environmental Pollution Control and Management, Institute of Eco-environmental and Soil Sciences, Guangdong Academy of Sciences, Guangzhou, 510650, China; Department of Applied Physics and Chemical Engineering, Tokyo University of Agriculture and Technology, Tokyo, 184-8588, Japan; National–Regional Joint Engineering Research Center for Soil Pollution Control and Remediation in South China, Guangdong Key Laboratory of Integrated Agro–environmental Pollution Control and Management, Institute of Eco-environmental and Soil Sciences, Guangdong Academy of Sciences, Guangzhou, 510650, China; School of Environment, Guangdong Provincial Key Laboratory of Chemical Pollution and Environmental Safety & MOE Key Laboratory of Theoretical Chemistry of Environment, South China Normal University, Guangzhou, 510006, China; Southern Marine Science and Engineering Guangdong Laboratory (Guangzhou), Guangzhou, 511458, China; National–Regional Joint Engineering Research Center for Soil Pollution Control and Remediation in South China, Guangdong Key Laboratory of Integrated Agro–environmental Pollution Control and Management, Institute of Eco-environmental and Soil Sciences, Guangdong Academy of Sciences, Guangzhou, 510650, China; National–Regional Joint Engineering Research Center for Soil Pollution Control and Remediation in South China, Guangdong Key Laboratory of Integrated Agro–environmental Pollution Control and Management, Institute of Eco-environmental and Soil Sciences, Guangdong Academy of Sciences, Guangzhou, 510650, China; National–Regional Joint Engineering Research Center for Soil Pollution Control and Remediation in South China, Guangdong Key Laboratory of Integrated Agro–environmental Pollution Control and Management, Institute of Eco-environmental and Soil Sciences, Guangdong Academy of Sciences, Guangzhou, 510650, China; Southern Marine Science and Engineering Guangdong Laboratory (Guangzhou), Guangzhou, 511458, China; National–Regional Joint Engineering Research Center for Soil Pollution Control and Remediation in South China, Guangdong Key Laboratory of Integrated Agro–environmental Pollution Control and Management, Institute of Eco-environmental and Soil Sciences, Guangdong Academy of Sciences, Guangzhou, 510650, China; National–Regional Joint Engineering Research Center for Soil Pollution Control and Remediation in South China, Guangdong Key Laboratory of Integrated Agro–environmental Pollution Control and Management, Institute of Eco-environmental and Soil Sciences, Guangdong Academy of Sciences, Guangzhou, 510650, China; School of Environment, Guangdong Provincial Key Laboratory of Chemical Pollution and Environmental Safety & MOE Key Laboratory of Theoretical Chemistry of Environment, South China Normal University, Guangzhou, 510006, China; State Key Laboratory of Freshwater Ecology and Biotechnology, Key Laboratory of Lake and Watershed Science for Water Security, Institute of Hydrobiology, Chinese Academy of Sciences, Wuhan, 430072, China; State Key Laboratory of Environmental Criteria and Risk Assessment, Chinese Research Academy of Environmental Sciences, Beijing, 100012, China

**Keywords:** nitrous oxide, clade III *nosZ,* lake sediments, microbial community

## Abstract

Nitrous oxide-reducing bacteria (N_2_ORB) play a pivotal role in regulating N_2_O emissions in aquatic ecosystems, with clade I and clade II *nosZ*-harboring microorganisms representing well-recognized contributors to microbial N_2_O consumption. Beyond conventional N_2_ORB, the recently identified clade III *nosZ* from soil may represent a previously overlooked potential N_2_O sink, yet the distribution and characterization remain largely unexplored in aquatic ecosystems. Here we established microcosm systems using sediments from five lakes and subjected them to warming temperature gradients to investigate the diversity and genomic characteristics of N_2_ORB. Hidden Markov model (HMM)-based analyses identified a total of 45 nonredundant *nosZ* sequences, including 12 affiliated with clade III *nosZ*. Clade III *nosZ* accounted for 10.2%–40.6% of total *nosZ* genes, indicating that clade III *nosZ*-harboring N_2_ORB is widespread and non-negligible. Reconstruction of metagenome-assembled genomes (MAGs) identified four phylogenetically novel clade III *nosZ*-harboring N_2_ORB, with these MAGs showing low average amino acid identity to their closest known reference genomes. These MAGs showed different denitrification gene inventories, with MAG33 lacking identifiable genes for upstream N_2_O-producing steps, suggesting a potential non-denitrifying N_2_O reducer. They also encoded oxygen-related stress-response genes, suggesting a potential ability to perform N_2_O respiration in the presence of oxygen. Unlike canonical clade I/II *nosZ* clusters, clade III *nosZ*-harboring MAGs lacked typical accessory genes and instead exhibited distinct neighboring transporter- and cytochrome-related genes. Together, our results provide evidence for the occurrence of clade III *nosZ*-harboring N_2_ORB in non-soil ecosystems and expand current understanding of their genomic traits.

## Introduction

Nitrous oxide (N_2_O) is a potent greenhouse gas that depletes stratospheric ozone and contributes to global warming [1, 2]. The primary biological sink for N_2_O is reduction to dinitrogen (N_2_), which is mediated by N_2_O-reducing bacteria (N_2_ORB) via N_2_O-reductase (NosZ) encoded by the *nosZ* gene [3, 4]. N_2_ORB were initially classified into two clades (clade I and clade II) [3, 5] based on amino acid sequence divergence. These clades often differ in host ecophysiology, including the co-occurrence of upstream denitrification genes and their potential roles as complete denitrifiers or non-denitrifying N_2_O reducers. A recent study identified a previously unrecognized protein family in soil, namely, the lactonase-type *nosZ* clade (clade III *nosZ*) [6], greatly expanding the known diversity of N_2_ORB. Clade III *nosZ* sequences differ markedly from the typical *nosZ* clades, yet retain structural features associated with N_2_O reduction. Therefore, exploring clade III *nosZ*-harboring N_2_ORB is critical for uncovering previously unrecognized N_2_O sink pathways and refining our understanding of N_2_O cycling in ecosystems.

Lake ecosystems are important components of the global nitrogen cycle, and N_2_O emissions have received considerable attention [7, 8]. The diversity and activity of clade I and clade II *nosZ*-harboring N_2_ORB in aquatic environments have been relatively well documented [9, 10]. In contrast, the recently identified clade III *nosZ*, also referred to as lactonase-type *nosZ*, has been reported mainly from soil, groundwater, and marine environments [6], while its diversity, abundance, host taxonomy, and genomic traits in lake ecosystems remain unclear. Owing to the absence of upstream denitrification genes [11], some N_2_ORB are incapable of nitrate/nitrite reduction and therefore act as net sinks for N_2_O [12]. However, the limited understanding of these N_2_ORB likely leads to an underestimation of the N_2_O sink capacity in aquatic systems [13]. Therefore, a comprehensive characterization of clade III *nosZ*-harboring N_2_ORB is essential to better constrain their ecological role and potential contribution to N_2_O mitigation. Here, we employed metagenomic approaches to investigate the diversity, phylogeny, and genomic potential of N_2_ORB in laboratory-incubated lake sediments. By combining hidden Markov model (HMM)-based identification with metagenome-assembled genome (MAG) reconstruction, we aimed to (i) characterize the distribution of different types of N_2_ORB, with a particular focus on clade III *nosZ*, and (ii) evaluate the genotypes of clade III *nosZ*-harboring N_2_ORB. Our findings provide genomic evidence for previously overlooked clade III *nosZ* diversity in lake sediments and establish a basis for future studies assessing their activity and ecological relevance in freshwater N_2_O cycling.

## Results and discussion

**Figure 1:**
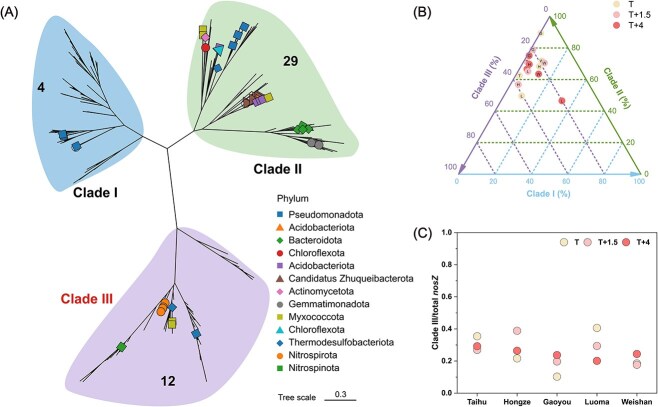
Phylogeny and temperature responses of *nosZ* clade in lake sediments. (A) Maximum-likelihood phylogenetic tree of *nosZ* genes, constructed using a combination of HMM searches and motif-based filtering, together with reference *nosZ* sequences. (B) Ternary phase diagram showing the relative abundance distribution of Clade I, Clade II, and Clade III *nosZ* across three temperature incubation treatments (T, T + 1.5, and T + 4). Each axis represents the relative abundance (%) of the respective clade. For any point in the ternary phase diagram, the three values sum to 100%. (C) The clade III/total *nosZ* ratio across five lakes under different treatments.

To assess the diversity of N_2_ORB in laboratory-incubated lake sediments (Texts S1 and S2), we constructed an HMM based on 264 reference NosZ sequences (including clade I, II, and III *nosZ*) to identify *nosZ*-encoding sequences in the assembled contigs [6] (Text S3). In total, 45 unique *nosZ* sequences were recovered, including 29 affiliated with the clade II *nosZ* and 12 with the clade III *nosZ* (Fig. 1A). Taxonomically, clade III *nosZ* genes were affiliated with the phyla *Nitrospirota*, *Nitrospinota*, *Pseudomonadota*, and *Myxococota*. In comparison, clade II *nosZ* genes showed broader taxonomic diversity across the seven phyla, whereas clade I *nosZ* genes were affiliated with *Pseudomonadota*. The mean abundances of clades I, II, and III *nosZ* were 3.33, 28.95, and 11.26 TPM, respectively (Fig. S1). Although clade II *nosZ* genes predominated across lakes, clade III *nosZ* genes accounted for 10.2%–40.6% of the combined abundance of all *nosZ* genes (Fig. 1B–C), indicating that clade III *nosZ*-harboring N_2_ORB are widespread and non-negligible members that contribute to N_2_O consumption. Despite the distinct warming scenarios among the lakes, warming did not significantly restructure the *nosZ*-containing community (Fig. S2), suggesting that the N_2_ORB community composition is relatively stable under current warming conditions.

**Figure 2:**
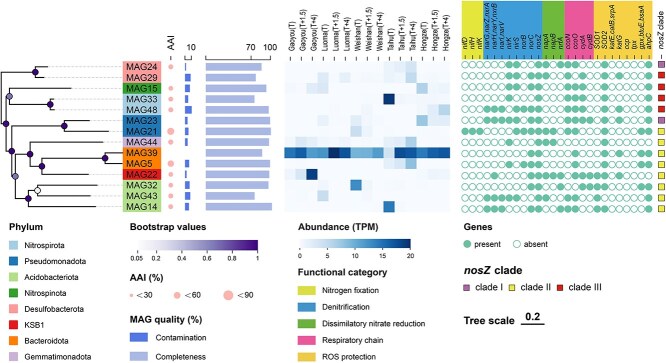
Characterization of reconstructed *nosZ*-harboring MAGs. Phylogenetic tree of the MAGs and their closest neighbors based on the analysis of 50 marker genes. Quality metrics, including contamination and completeness, are shown together with AAI (%) values calculated using TypeMat in MiGA Online by comparing each MAG against reference genomes. Abundance and gene inventories of the *nosZ*-harboring MAGs are displayed. The different colors of the MAGs represent the corresponding phylum. The heatmap illustrates MAG abundance across samples and temperature treatments. Filled circles indicate detected genes, whereas unfilled circles indicate undetected genes. Detailed gene information is provided in Supplementary Information Table S3.

To dissect the genotypes of specific N_2_ORB, MAGs were reconstructed from the sediment samples using MetaBAT2 [14] (Text S4; Fig. 2). HMM-based classification of *nosZ* sequences identified 14 MAGs as putative N_2_ORB [6]. All MAGs exhibited estimated contamination below 10% and completeness above 70% (Table S1). Among them, four MAGs (MAG15, MAG29, MAG33, and MAG48) harbored the clade III *nosZ* gene and were assigned to the phyla *Nitrospinota, Desulfobacterota*, *Nitrospirota,* and *Nitrospirota,* respectively (Fig. S3). These phyla have previously been reported to contain clade III *nosZ* mainly in soil, groundwater, and marine environments [6]. To the best of our knowledge, this is the first evidence of clade III *nosZ*-harboring MAGs in lake ecosystems. Compared with their closest known relatives, all four MAGs harboring the clade III *nosZ* gene showed amino acid identity (AAI) values less than 50%, suggesting that they represent previously uncharacterized clade III *nosZ*-harboring N_2_ORB lineages (Table S2). MAG48 encoded a near-complete denitrification gene set, whereas MAG15, MAG29, and MAG33 lacked detectable *nar*/*nxr*-related genes, suggesting limited potential for nitrate reduction. Notably, the nitric oxide reductase genes *norB*/*norC* were not detected in MAG33, suggesting that it may primarily function in N_2_O consumption rather than contribute to N_2_O production. This finding expands the diversity of non-denitrifying N_2_ORB, which have previously been predominantly reported within clade II *nosZ* [4]. However, due to the incomplete recovery of MAG33 (72.4% completeness), the absence of *norB*/*norC* may reflect genome incompleteness or true absence, warranting further validation. Additionally, all clade III *nosZ-*harboring MAGs carried *ccoN*, *cydA*, *SOD2*, and *ahpC*, suggesting that they are capable of microaerobic respiration and possess mechanisms to tolerate oxidative stress [15, 16]. We also observed that eight MAGs (MAG5, MAG14, MAG21, MAG22, MAG32, MAG39, MAG43, and MAG44) contained the clade II *nosZ* gene but lacked at least one denitrification gene. In addition, two MAGs (MAG23 and MAG24) harboring the clade I *nosZ* gene, of which MAG23 possessed a complete denitrification pathway. These findings highlight clade III *nosZ*-harboring N_2_ORB as a previously unrecognized and potentially important component of N_2_ORB.

A gene-cluster analysis of 14 *nosZ*-containing MAGs (Fig. S4) showed that clade I *nosZ*-harboring MAGs and a subset of clade II *nosZ*-harboring MAGs possess conserved accessory-gene modules associated with canonical *nosZ*, including *nosR*, *nosD*, *nosF*, *nosY*, and *nosL*. In addition, many clade II *nosZ*-harboring MAGs encode a putative *nosB* [4, 6, 12]. In contrast, clade III *nosZ*-harboring MAGs lacked identifiable canonical *nos* accessory genes. Instead, genes encoding cytochromes and transporter-related proteins were found in the genomic vicinity of the clade III *nosZ*. In the clade III *nosZ*-harboring MAG15 and MAG29, two adjacent genes encode an ABC transporter ATP-binding protein and a permease that show sequence similarity to canonical NosF and NosY, respectively. However, these genes were located at a substantial distance from *nosZ* and were not accompanied by NosD or NosL. In light of the recently described clade III/L-*nosZ* architecture, such dispersed transporter genes should be described as NosF/NosY-like homologues rather than annotated as canonical NosF or NosY [6]. Whether these genes contribute to N_2_O reduction in these MAGs remains to be experimentally validated.

In summary, this study provides evidence for the occurrence of clade III *nosZ*-harboring N_2_ORB in a non-soil environment [6], expanding their known distribution beyond previously reported habitats. We recovered previously unrecognized clade III *nosZ*-harboring N_2_ORB from sediment-derived microcosms inoculated with lakes, suggesting that this overlooked lineage may also be present in other yet underexplored ecosystems (e.g. aquatic and engineering systems). These findings further broaden our current understanding of the diversity and ecological distribution of clade III *nosZ*-harboring N_2_ORB. Notably, the detection of non-denitrifying clade III *nosZ*-harboring N_2_ORB suggests that this lineage may represent a previously underestimated component of potential N_2_O sink. However, their *in situ* distribution, functional activity, and ecological relevance remain to be confirmed. In particular, the contribution of clade III *nosZ*-harboring N_2_ORB to N_2_O mitigation in natural environments requires further investigation, as their role may have been long overlooked in N_2_O cycling. Therefore, further investigation of their genotypic and phenotypic traits is needed to better constrain their contribution to the N_2_O sink.

## Supplementary Material

Supplementary_material_ycag194

## Data Availability

The raw metagenomic sequencing data have been deposited in the NCBI database accession number PRJNA1459056.
