# Supplementary material for: Unexpected novel clade III type nitrous oxide-reducing bacteria from incubated lake sediments
Source: ISME Commun. 2026 Jul 5;6(1):ycag194. doi: 10.1093/ismeco/ycag194 (PMC13477213; doi:10.1093/ismeco/ycag194)
Supplement: Supplementary file 1 [file supplementary_material_ycag194.docx]

**Supporting Information**

**Unexpected novel clade III type nitrous oxide-reducing bacteria from incubated lake sediments**

Siyu Wang^1,3,§^, Fei Shui^1,2,5,§^, Yiwen Zhou^1,*^, Xiao Wang^1^, Yuli Zeng^1,5^, Yinghao Shan^1^, Jining Li^1^, Liguo Zhang^2^, Kang Song^4^, Fengchang Wu^6^

^1^ National–Regional Joint Engineering Research Center for Soil Pollution Control and Remediation in South China, Guangdong Key Laboratory of Integrated Agro–environmental Pollution Control and Management, Institute of Eco-environmental and Soil Sciences, Guangdong Academy of Sciences, Guangzhou 510650, China

^2^ School of Environment, Guangdong Provincial Key Laboratory of Chemical Pollution and Environmental Safety & MOE Key Laboratory of Theoretical Chemistry of Environment, South China Normal University, Guangzhou, 510006, China

^3^ Department of Applied Physics and Chemical Engineering, Tokyo University of Agriculture and Technology, Tokyo 184-8588, Japan

^4^ State Key Laboratory of Freshwater Ecology and Biotechnology, Key Laboratory of Lake and Watershed Science for Water Security, Institute of Hydrobiology, Chinese Academy of Sciences, Wuhan 430072, China

^5^ Southern Marine Science and Engineering Guangdong Laboratory (Guangzhou), Guangzhou, China

^6^ State Key Laboratory of Environmental Criteria and Risk Assessment, Chinese Research Academy of Environmental Sciences, Beijing 100012, China

^§^ These authors contributed equally.

^*^ Corresponding author.

E-mail address: ahzhoull@hotmail.com (Y. Zhou)

**Supplementary Methods**

**Text S1 Sampling and experimental setup**

Sediment samples were collected from five lakes in China in July 2023, and placed in sterile plastic bags. Subsequently, they were stored in coolers away from light, and transported to the laboratory as soon as possible (see reference [1] for detailed sampling information). After removing large particles and plant debris, the sediments were thoroughly homogenized and transferred into the experimental apparatus.

The incubation system adopted a nested experimental design, consisting of an outer culture tank and an internal experimental jar. Each jar was filled with sediment and overlying water at a volume ratio of 2:3. Incubation temperature was regulated using a temperature-control device installed in the outer culture tank. Three temperature treatments were established, each with three replicates: a control treatment (T), in which the water temperature was not artificially increased and fluctuated with ambient air temperature in Guangzhou, China; a +1.5 ℃ warming treatment (T+1.5); and a +4 ℃ warming treatment (T+4).

The incubation was performed under anoxic conditions to simulate natural lake environments. Dissolved oxygen (DO) was continuously monitored for 120 min using a dissolved oxygen meter (FireSting-GO2，PyroScience GmbH，Aachen，Germany). After the DO reading had stabilized, measurements were first taken at the air-water interface. The probe was then slowly lowered to the middle of the water column and, after stabilization, further inserted 0.5 cm into the sediment surface. The DO concentration at the sediment surface was 0, confirming that anaerobic conditions had been established during incubation (Fig. S5).

Throughout the two-year experiment, deionized water was periodically added to compensate for water loss caused by experimental manipulation and evaporation, thereby maintaining the steady-state balance of the microcosms. Ammonium chloride and sodium nitrate were supplied as nitrogen sources, while glucose was added as a carbon source. The concentrations and combinations of the added nitrogen and carbon sources varied among lakes, as shown in Table S4.

**Text S2 DNA extraction and sequencing**

Biomass samples were collected to analyze the microbial community. Total DNA was extracted from the samples using the E.Z.N.A. soil DNA Kit (Omega Bio-tek) according to the manufacturer’s procedure with the following modifications. Shotgun metagenomic sequencing libraries were constructed and sequenced by Biozeron Biological Technology (Shanghai, China). Briefly, 1 μg of genomic DNA from each sample was fragmented using a Covaris S220 focused ultrasonicator (Woburn, MA, USA), and sequencing libraries were prepared with an average insert size of approximately 450 bp. All libraries were sequenced on an MGI-T7 platform in paired-end 150 bp mode. Approximately 15 Gb of raw sequencing data were generated per sample.

The paired-end Illumina reads were trimmed of adaptors, and low-quality reads (those with a length < 50 bp, a quality value < 20 or N bases) were removed by Fastp[2] v0.23.2. Metagenomic data were assembled using MEGAHIT[3] v1.2.9. Contigs with a length ≥ 500 bp were selected as the final assembly result. The coding sequence (CDS) from each assembled contig was predicted using Prodigal[4] v2.6.3. A nonredundant gene catalog with 90% sequence identity and 95% coverage was constructed using CD-HIT[5] v4.8.1. For each sample, quality-controlled reads were mapped to the nonredundant gene catalog using SOAPaligner[6] vsoap2.21 release with a minimum identity of 95%. Taxonomic annotation was conducted using Kraken2 (standard database v20260226) with default parameters. Functional annotations were obtained by DIAMOND[7] v2.1.6 against eggNOG database. Gene abundance was then quantified using CoverM[8] v0.7.06. Abundance values were reported as TPM (transcripts per million), representing the length- and library-size-normalized relative gene abundance.

**Text S3 Identification of candidate *nosZ* genes**

Candidate *nosZ* reads identified by ROCker were further validated using hidden Markov model (HMM). Specifically, the ROCker tool was used to identify sequence reads carrying *nosZ* in the samples[9]. Then, putative NosZ sequences were then identified from the predicted protein dataset using a combined approach of HMM searching and motif‑based filtering[10]. An HMM profile built for NosZ was employed with HMMER v3.4 (http://hmmer.org/) to screen the non‑redundant protein sequences. Candidate hits with an E‑value below 10^−5^ were retained and further filtered by conserved motif sequences, with the exclusion of the DXHH pattern, to ensure high confidence in the final assignment. Retained candidate sequences were aligned with reference NosZ sequences using MAFFT[11] v7.526 with the ‑auto parameter. A maximum-likelihood phylogenetic tree was then generated using FastTree[12] v2.1.11 with default settings. Candidate sequences were assigned to Clade III NosZ when they clustered with reference Clade III NosZ sequences and showed strong phylogenetic support.

**Text S4 Reconstruction and analyses of metagenome-assembled genomes (MAGs)**

Contigs with a length of at least 1,000 bp were selected for further binning to obtain metagenome-assembled genomes (MAGs). Multiple binning tools, including MetaBAT[13] v2.12.1, CONCOCT[14] v0.5.0, and MaxBin2[15] v2.2.5, were applied to group the contigs. The resulting bins were then integrated and refined using DAS_Tool[16] v1.1.0 to produce consolidated bins, which were subsequently purified with RefineM[17] v0.0.24 and reclassified as MAGs. All MAGs were combined and subjected to redundancy removal using dRep[18] v2.2.9 through a two‑step clustering process: first, primary clusters were formed at ≥ 90% average nucleotide identity (ANI) estimated by Mash; second, within each primary cluster, secondary clustering was performed at ≥ 99% ANI with at least 10% genome overlap. The completeness, contamination, and strain heterogeneity of the final MAGs were evaluated using CheckM[19] v1.2.2, and only those with more than 70% completeness and less than 10% contamination were retained for downstream analyses. Functional annotation of the retained MAGs was performed using the eggNOG database to assign orthologous groups and predict gene functions. The amino acid identity (AAI) of the MAGs was determined on the basis of the reference genome using TypeMat on MiGA Online (https://aau.microbial-genomes.org/) to determine the novelty of the MAGs. Novel species were generally considered those with an AAI ≤ 95%. Taxonomic classification of metagenome-assembled genomes (MAGs) was performed using GTDB-Tk[20] (Genome Taxonomy Database Toolkit, release 226) with the classify_wf workflow. Default parameters were applied. For phylogenetic analysis, a genome tree was inferred using the infer workflow in GTDB-Tk based on the concatenated bacterial marker gene alignment (bac120).

**Supplementary figures**

**Figure S1 Gene abundances of clades I, II, and III *nosZ* in lake sediments.**

**Figure S2 Alpha and beta diversity of *nosZ* gene communities across temperature treatments.** (A) Shannon and (B) Simpson diversity indices of *nosZ* gene communities under the three temperature treatments (T, T+1.5, and T+4). One-way ANOVA showed no significant differences in either alpha diversity metric among treatments (*P* > 0.05). (C) Principal coordinate analysis (PCoA) of weighted UniFrac distances showing beta diversity patterns of *nosZ* gene communities across temperature treatments. Colors indicate temperature treatments, and ellipses represent 95% confidence intervals. PERMANOVA detected no significant effect of temperature on *nosZ* community structure (F = 0.578, *P* = 0.913).

**Figure S3 NosZ phylogeny derived from metagenome-assembled genomes (MAGs).** The phylogenetic tree was constructed using NosZ amino acid sequences identified by HMM searches, together with reference NosZ sequences. The gray squares represent reference sequences. The scale bar represents 0.3 substitutions per site.

**Figure S4 Nos gene cluster-based analyses of 14 MAGs.** Distinct genes within the clusters are color-coded. Genes not directly related to denitrification are shown in white and labeled as “other”.

**Figure S5 Changes in dissolved oxygen (DO) concentrations at different depths.**

Supplementary Table**s**

**Table S1 Properties and taxonomic assignments of MAGs.**

| **MAG ID** | **Completeness (%)** | **Contamination (%)** | **Taxonomy** |
| --- | --- | --- | --- |
| MAG5 | 95.48 | 5.30 | order *Chitinophagales* |
| MAG14 | 98.29 | 5.13 | phylum *Acidobacteriota* |
| MAG15 | 89.96 | 6.32 | phylum *Nitrospinota* |
| MAG21 | 96.80 | 4.45 | order *Burkholderiales* |
| MAG22 | 94.44 | 2.01 | phylum *Methylomirabilota* |
| MAG23 | 95.56 | 3.24 | Unclassified |
| MAG24 | 82.91 | 1.29 | class *Desulfuromonadia* |
| MAG29 | 74.39 | 7.34 | Unclassified |
| MAG32 | 93.16 | 5.95 | phylum *Acidobacteriota* |
| MAG33 | 72.35 | 4.12 | phylum *Thermodesulfobacteriota* |
| MAG39 | 83.86 | 0.00 | family *Chitinophagaceae* |
| MAG43 | 72.53 | 6.84 | class *Vicinamibacteria* |
| MAG44 | 93.96 | 3.30 | class *Gemmatimonadia* |
| MAG48 | 93.64 | 8.78 | phylum *Thermodesulfobacteriota* |

**Table S2** **Analysis of MAGs with the Microbial Genome Atlas (MiGA).** Listed for each MAG is the closest relative, the Genome accession number, and the average amino acid identity (AAI) of each MAG.

| **MAG ID** | **Closest relative** | **Genome accession number** | **AAI (%)** |
| --- | --- | --- | --- |
| MAG5 | *Paraflavitalea devenefica* | GCF 011759375 1 | 58.23 |
| MAG14 | *Mesoterricola sediminis* | GCF 030295425 1 | 42.71 |
| MAG15 | *Nitrospina watsonii* | GCF 946900835 1 | 42.29 |
| MAG21 | *Aquabacterium humicola* | GCF 030167915 1 | 62.94 |
| MAG22 | *Methylomirabilis oxygeniifera asm* | GCA 000091165 1 | 42.26 |
| MAG23 | Unclassified | - | - |
| MAG24 | *Trichlorobacter lovleyi* SZ | GCF 000020385 1 | 45.43 |
| MAG29 | Unclassified | - | - |
| MAG32 | *Candidatus Gilichinskyi gelida* | GCA 038040385 1 | 42.30 |
| MAG33 | *Trichlorobacter lovleyi* SZ | GCF 000020385 1 | 43.75 |
| MAG39 | Unclassified | - | - |
| MAG43 | *Luteitalea pratensis* | GCF 001618865 1 | 45.39 |
| MAG44 | *Gemmatirosa kalamazoonensis* | GCF 000522985 1 | 45.95 |
| MAG48 | *Trichlorobacter lovleyi* SZ | GCF 000020385 1 | 44.76 |

**Table S3 Information on microbial functional genes involved in nitrogen metabolism，respiratory chain, and oxidative stress defense identified in this study.**

| **Functional category** | **KEGG number** | **Gene name** | **Encoded protein [EC]** |
| --- | --- | --- | --- |
| Nitrogen fixation | K02586 | *nifD* | nitrogenase molybdenum-iron protein alpha chain [EC:1.18.6.1] |
|  | K02588 | *nifH* | nitrogenase iron protein NifH |
|  | K02591 | *nifK* | nitrogenase molybdenum-iron protein beta chain [EC:1.18.6.1] |
| Denitrification | K00370 | *narG, narZ, nxrA* | nitrate reductase/nitrite oxidoreductase, alpha subunit [EC:1.7.5.1 1.7.99.-] |
|  | K00371 | *narH, narY, nxrB* | nitrate reductase/nitrite oxidoreductase, beta subunit [EC:1.7.5.1 1.7.99.-] |
|  | K00374 | *narI, narV* | nitrate reductase gamma subunit [EC:1.7.5.1 1.7.99.-] |
|  | K00368 | *nirK* | nitrite reductase (NO-forming) [EC:1.7.2.1] |
|  | K15864 | *nirS* | nitrite reductase (NO-forming) [EC:1.7.2.1 1.7.99.1] |
|  | K04561 | *norB* | nitric oxide reductase subunit B [EC:1.7.2.5] |
|  | K02305 | *norC* | nitric oxide reductase subunit C |
|  | K00376 | *nosZ* | nitrous-oxide reductase [EC:1.7.2.4] |
| Dissimilatory  nitrate reduction | K02567 | *napA* | periplasmic nitrate reductase [EC:1.9.6.1] |
|  | K02568 | *napB* | periplasmic nitrate reductase, electron transfer subunit |
|  | K03385 | *nrfA* | nitrite reductase (cytochrome c-552) [EC:1.7.2.2] |
| Respiratory chain | K00404 | *ccoN* | cytochrome cbb3 oxidase subunit I [EC:7.1.1.9] |
|  | K00405 | *ccoO* | cytochrome cbb3 oxidase subunit II |
|  | K00425 | *cydA* | cytochrome bd ubiquinol oxidase subunit I [EC:7.1.1.7] |
|  | K00426 | *cydB* | cytochrome bd ubiquinol oxidase subunit II [EC:7.1.1.7] |
| ROS  protection | K04565 | *SOD1* | superoxide dismutase, Cu-Zn family [EC:1.15.1.1] |
|  | K04564 | *SOD2* | superoxide dismutase, Fe-Mn family [EC:1.15.1.1] |
|  | K03781 | *katE, catB, srpA* | catalase [EC:1.11.1.6] |
|  | K03782 | *katG* | catalase-peroxidase [EC:1.11.1.21] |
|  | K00428 | *ccp* | cytochrome c peroxidase [EC:1.11.1.5] |
|  | K11065 | *tpx* | thiol peroxidase |
|  | K00432 | *gpx, btuE, bsaA* | glutathione peroxidase |
|  | K03386 | *ahpC* | alkyl hydroperoxide reductase |

**Table S4 Target final concentrations of added nitrogen sources and carbon source in the overlying water.**

| **Lake** | **NO_3_^−^-N (mg L^−1^)** | **NH_4_^+^-N (mg L^−1^)** | **DOC (mg L^−1^)** |
| --- | --- | --- | --- |
| Taihu | 3.00 | 1.00 | 8.00 |
| Hongze | 1.00 | 1.00 | 6.00 |
| Gaoyou | 0.50 | 0.50 | 6.00 |
| Luoma | 0.10 | 0.50 | 6.00 |
| Weishan | - | 0.20 | 6.00 |

**References**

1.	Song K, Wang S, Xu X , Ma J, Yang Y, Zeng Y *et al.* Benthic clade II-type nitrous oxide reducers suppress nitrous oxide emissions in shallow lakes. *Sci Total Environ*. 2024;**931**:172908 <https://doi.org/10.1016/j.scitotenv.2024.172908>

2.	Chen S, Zhou Y, Chen Y, Gu J*.* Fastp: an ultra-fast all-in-one fastq preprocessor. *Bioinformatics*. 2018;**34**:i884-i90 <https://doi.org/10.1093/bioinformatics/bty560>

3.	Li D, Liu C-M, Luo R, Sadakane K, Lam T-W*.* Megahit: an ultra-fast single-node solution for large and complex metagenomics assembly via succinct de bruijn graph. *Bioinformatics*. 2015;**31**:1674-76 <https://doi.org/10.1093/bioinformatics/btv033>

4.	Hyatt D, Chen G-L, LoCascio PF, Land ML, Larimer FW, Hauser LJ*.* Prodigal: prokaryotic gene recognition and translation initiation site identification. *BMC Bioinformatics*. 2010;**11**:119 <https://doi.org/10.1186/1471-2105-11-119>

5.	Fu L, Niu B, Zhu Z, Wu S, Li W*.* Cd-hit: accelerated for clustering the next-generation sequencing data. *Bioinformatics*. 2012;**28**:3150-2 <https://doi.org/10.1093/bioinformatics/bts565>

6.	Li R, Li Y, Kristiansen K , Wang J*.* Soap: short oligonucleotide alignment program. *Bioinformatics*. 2008;**24**:713-14 <https://doi.org/10.1093/bioinformatics/btn025>

7.	Buchfink B, Xie C, Huson DH. Fast and sensitive protein alignment using diamond. *Nat Methods*. 2015;**12**:59-60 <https://doi.org/10.1038/nmeth.3176>

8.	Aroney STN, Newell RJP, Nissen JN, Camargo AP, Tyson GW, Woodcroft BJ*.* CoverM: read alignment statistics for metagenomics. *Bioinformatics*. 2025;**41**:btaf147 <https://doi.org/10.1093/bioinformatics/btaf147>

9.	Orellana LH, Rodriguez-R LM, Konstantinidis KT. Rocker: accurate detection and quantification of target genes in short-read metagenomic data sets by modeling sliding-window bitscores. *Nucleic Acids Res*. 2017;**45**:e14-e14 <https://doi.org/10.1093/nar/gkw900>

10.	He G, Wang W, Chen G, Xie Y, Parks JM, Davin ME *et al.* A novel bacterial protein family that catalyses nitrous oxide reduction. *Nature*. 2025;**646**:152-60 <https://doi.org/10.1038/s41586-025-09401-4>

11.	Rozewicki J, Li S, Amada KM, Standley DM, Katoh K*.* MAFFT-DASH: integrated protein sequence and structural alignment. *Nucleic Acids Res*. 2019;**47**:W5-W10 <https://doi.org/10.1093/nar/gkz342>

12.	Price MN, Dehal PS, Arkin AP. Fasttree 2 – approximately maximum-likelihood trees for large alignments. *PLOS ONE*. 2010;**5**:e9490 <https://doi.org/10.1371/journal.pone.0009490>

13.	Kang DD, Froula J, Egan R, Wang Z*.* MetaBat, an efficient tool for accurately reconstructing single genomes from complex microbial communities. *PeerJ*. 2015;**3**:e1165 <https://doi.org/10.7717/peerj.1165>

14.	Alneberg J, Bjarnason BS, de Bruijn I, Schirmer M, Quick J, Ijaz UZ *et al.* Binning metagenomic contigs by coverage and composition. *Nat Methods*. 2014;**11**:1144-46 <https://doi.org/10.1038/nmeth.3103>

15.	Wu YW, Simmons BA, Singer SW. Maxbin 2.0: an automated binning algorithm to recover genomes from multiple metagenomic datasets. *Bioinformatics*. 2016;**32**:605-07 <https://doi.org/10.1093/bioinformatics/btv638>

16.	Sieber CMK, Probst AJ, Sharrar A, Thomas BC, Hess M, Tringe SG *et al.* Recovery of genomes from metagenomes via a dereplication, aggregation and scoring strategy. *Nat Microbiol*. 2018;**3**:836-43 <https://doi.org/10.1038/s41564-018-0171-1>

17.	Parks DH, Rinke C, Chuvochina M, Chaumeil P-A, Woodcroft BJ, Evans PN *et al.* Recovery of nearly 8,000 metagenome-assembled genomes substantially expands the tree of life. *Nat Microbiol*. 2017;**2**:1533-42 <https://doi.org/10.1038/s41564-017-0012-7>

18.	Olm MR, Brown CT, Brooks B, Banfield JF*.* Drep: a tool for fast and accurate genomic comparisons that enables improved genome recovery from metagenomes through de-replication. *ISME J*. 2017;**11**:2864-68 <https://doi.org/10.1038/ismej.2017.126>

19.	Parks DH, Imelfort M, Skennerton CT, Hugenholtz P, Tyson GW*.* CheckM: assessing the quality of microbial genomes recovered from isolates, single cells, and metagenomes. *Genome Res*. 2015;**25**:1043-55 <https://doi.org/10.1101/gr.186072.114>

20.	Parks DH, Chuvochina M, Rinke C, Mussig AJ, Chaumeil P-A, Hugenholtz P*.* GTDB: an ongoing census of bacterial and archaeal diversity through a phylogenetically consistent, rank normalized and complete genome-based taxonomy. *Nucleic Acids Res.* 2022;**50**:D785-D94 <https://doi.org/10.1093/nar/gkab776>
